# That’s All She Wrote

**DOI:** 10.13023/jah.0401.02

**Published:** 2022-02-13

**Authors:** Charlotte S. Seidman

**Affiliations:** Seidman Writing

**Keywords:** Appalachia, writing, editing, career, nurse practice

## Abstract

With the publication of this issue, I am retiring and formally handing over the reins as managing editor of the *Journal of Appalachian Health* to Rachel Dixon. I have had a 50+ year career and have been blessed to have had the opportunity to meet and work with the best health professionals in preventive medicine and public health.

With the publication of this issue, I am retiring and formally handing over the reins as managing editor of the *Journal of Appalachian Health* to Rachel Dixon. I have had a 50+ year career and have been blessed to have had the opportunity to meet and work with the best health professionals in preventive medicine and public health. I’ve had the privilege to teach everything from Sunday School to graduate school and to serve our country as an Army Nurse in Vietnam.

The trajectory of my career arced from “nurse”—caring for critically ill patients; to “nurse practitioner”—caring for and teaching patients to take control of their own health; to “NP faculty”—teaching NPs to care for their patients; to “managing editor”—providing knowledge to scores of healthcare professionals through various books and journals.

This farewell letter is meant to recognize the mentors who helped form my personal and professional life, who recognized in me something that I often did not. My parents gave me both roots, in the wonderful Appalachian Mountains of central PA, and wings, to go out and explore the world and to secure my place.

Mrs. Velva Daihl was my first mentor, although I didn’t realize it at the time; she was my journalism teacher in high school. She taught me the power of words, that it matters how we use them, and that brevity and structure were important components of successful writing. I never imagined in 1964 that what she taught me would provide the foundation for much of my career.

Neil Vaughn took me “under his wing” as my preceptor through the NP program and internship at UC Davis. His gentle ways taught me more than the practice of family medicine; they taught me how to live my life as a caring human being.

In the Graduate School of Public Health at San Diego State University, I met the three most influential forces in my career: Doug Scutchfield, Abram (“Bud”) Benenson, and Kevin Patrick. From 1981 to 2021, I worked with these gentlemen in clinical practice and research, teaching, and writing and editing books and journals.^1^–^6^ They each had an influence in my growth as an editor. Kevin said it best:

My personal attraction is to the extraordinary magic involved in moving clusters of ideas and thoughts, often unformed and poorly organized, into coherent written form, and then processing this—with good editing (conceptual, contextual, copy)—to produce a final document of record for all to use.

Special thanks to Kevin and Doug for encouraging me to create a semester-long course on writing and editing for preventive medicine residents and for masters and doctoral PH students. I loved the challenge and was able to reach several hundred students through the course.

I thank all of these mentors for the guidance they provided me. My hope as I retire is that my career will have had a positive impact on those whose lives I’ve had the honor to care for, teach, and reach through the published word; that the circle of mentoring will have been somehow enriched by my contribution.
